# The Use of Eye-Movement Desensitization Reprocessing (EMDR) Therapy in Treating Post-traumatic Stress Disorder—A Systematic Narrative Review

**DOI:** 10.3389/fpsyg.2018.00923

**Published:** 2018-06-06

**Authors:** Gemma Wilson, Derek Farrell, Ian Barron, Jonathan Hutchins, Dean Whybrow, Matthew D. Kiernan

**Affiliations:** ^1^Department of Nursing, Midwifery and Health, Faculty of Health and Life Sciences, Northumbria University, Newcastle upon Tyne, United Kingdom; ^2^Department of Psychology, Institute of Health & Society, University of Worcester, Worcester, United Kingdom; ^3^School of Education and Social Work, University of Dundee, Dundee, United Kingdom; ^4^Hutchins Psychology Services ltd, St Albans, United Kingdom; ^5^School of Healthcare Sciences, College of Biomedical and Life Sciences, Cardiff University, Cardiff, United Kingdom

**Keywords:** eye movement desensitization and reprocessing (EMDR), EMDR therapy, trauma exposure, post-traumatic stress disorder, PTSD, review

## Abstract

**Aim:** There is an extensive body of research examining the efficacy of Eye-Movement Desensitization Reprocessing (EMDR) therapy in treatment of Post-traumatic Stress Disorder (PTSD). This systematic narrative review aimed to systematically, and narratively, review robust evidence from Randomized-Controlled Trials examining the efficacy of EMDR therapy.

**Method:** Eight databases were searched to identify studies relevant to the study aim. Two separate systematic searches of published, peer-reviewed evidence were carried out, considering relevant studies published prior to April 2017. After exclusion of all irrelevant, or non-robust, studies, a total of two meta-analyses and four Randomized-Controlled Trials were included for review.

**Results:** Data from meta-analyses and Randomized-Controlled Trials included in this review evidence the efficacy of EMDR therapy as a treatment for PTSD. Specifically, EMDR therapy improved PTSD diagnosis, reduced PTSD symptoms, and reduced other trauma-related symptoms. EMDR therapy was evidenced as being more effective than other trauma treatments, and was shown to be an effective therapy when delivered with different cultures. However, limitations to the current evidence exist, and much current evidence relies on small sample sizes and provides limited follow-up data.

**Conclusions:** This systematic narrative review contributes to the current evidence base, and provides recommendations for practice and future research. This review highlights the need for additional research to further examine the use of EMDR therapy for PTSD in a range of clinical populations and cultural contexts.

## Introduction

Eye-Movement Desensitization Reprocessing (EMDR) is a form of Psychotherapy developed by Shapiro (1995). Ostensibly, EMDR therapy is a trans-diagnostic, integrative psychotherapy that has been extensively researched and there is a growing empirical base for effective for the treatment of adverse life experiences, namely Post-traumatic Stress Disorder (PTSD) (Farrell, 2016). EMDR therapy utilizes a theoretical framework of Adaptive Information Processing (AIP), which posits that the primary source of psychopathology is the presence of memories of adverse life experiences inadequately processed by the brain (Felitti et al., 1998). There is much evidence examining the use of EMDR therapy as a treatment for trauma, however, much of this evidence centers upon non-Randomized Controlled Trials (RCTs).

This report intends to systematically, and narratively, review robust RCT evidence examining the efficacy of EMDR therapy.

## Methods

**Figure 1:**
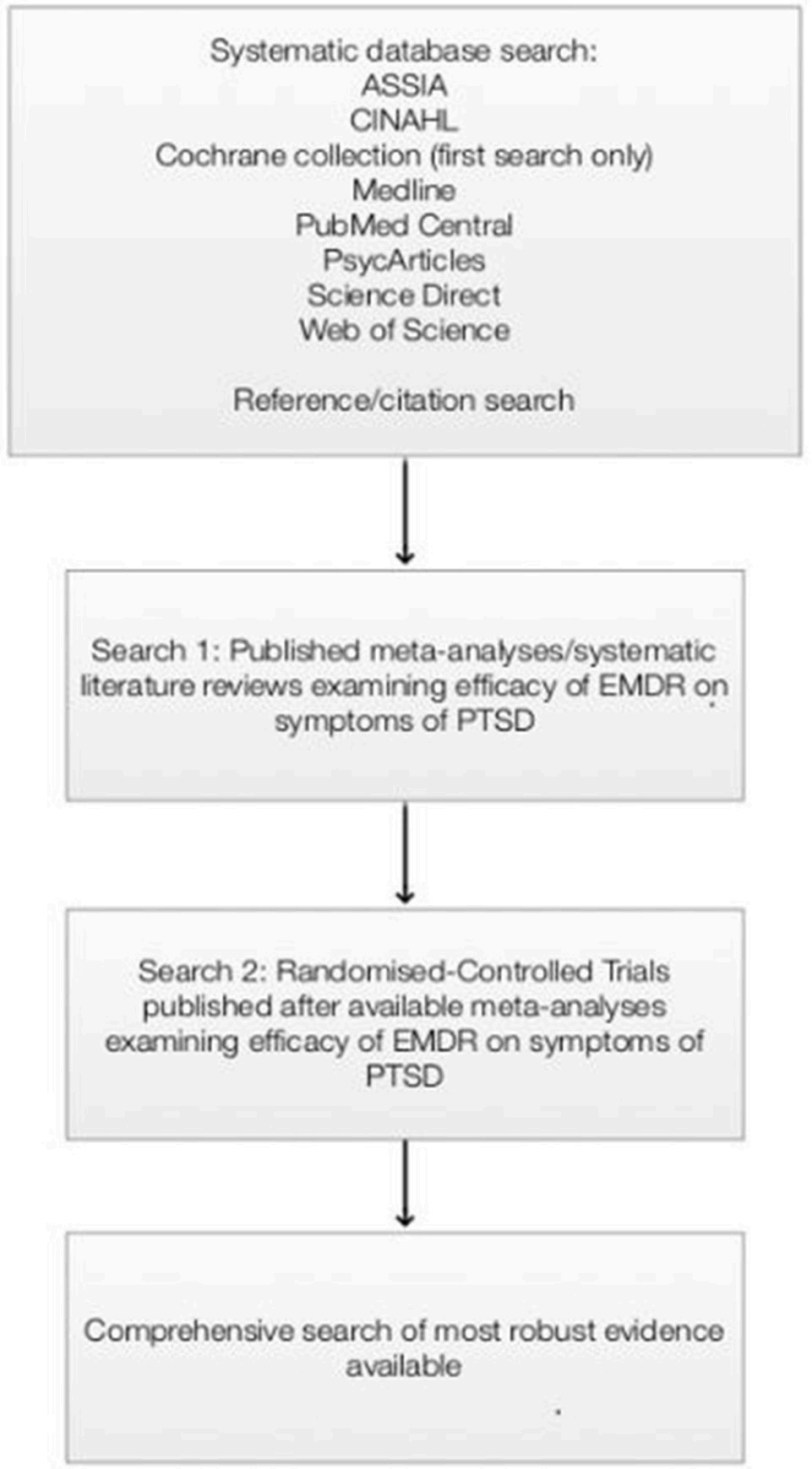
Systematic search process.

A systematic literature search of the databases was carried out, as outlined in Figure 1. After an initial scoping review of the literature, it became apparent that relevant meta-analyses of RCT studies were available. Therefore, the first systematic search gathered evidence of all systematic reviews and meta-analyses, which have synthesized and presented collective RCT evidence, examining the efficacy of EMDR therapy. All of the meta-analyses returned from this search specifically focused on the efficacy of EMDR therapy on PTSD symptoms - the most recent meta-analysis included papers prior to 2014. As a result, a second search was carried out to look at RCT studies investigating the efficacy of EMDR therapy on PTSD symptoms between 2014 and 2017, to ensure the most recent evidence was considered.

### Search 1

A database search of published peer-reviewed systematic evidence relevant to the aim of this review was carried out, considering all relevant papers prior to April 2017 (Table 1). All databases were accessed using Northumbria University library's online subscription.

**Table 1:** Search strategy utilized for both systematic searches.

Source	ASSIA CINAHL Cochrane library Medline PsycARTICLES Pubmed central Science Direct Freedom Collection Web of Science
Search field	ASSIA (AB Abstract) CINAHL (AB Abstract) Cochrane library (Title, abstract, keywords) Medline (AB Abstract) PsycARTICLES (AB Abstract) Pubmed central (Abstract) Science Direct Freedom Collection (Abstract, title, keywords) Web of Science (Title)
Language	English only
Exclusion	Non-English language Non-RCTs Non-peer reviewed papers Pilot studies/RCT protocol data Studies including children/adolescents only EMDR not focus of report
Search terms	(eye movement desensitization reprocessing OR EMDR) AND (systematic review OR meta-analysis)
Year of publication	All papers published prior to April 2017

**Figure 2:**
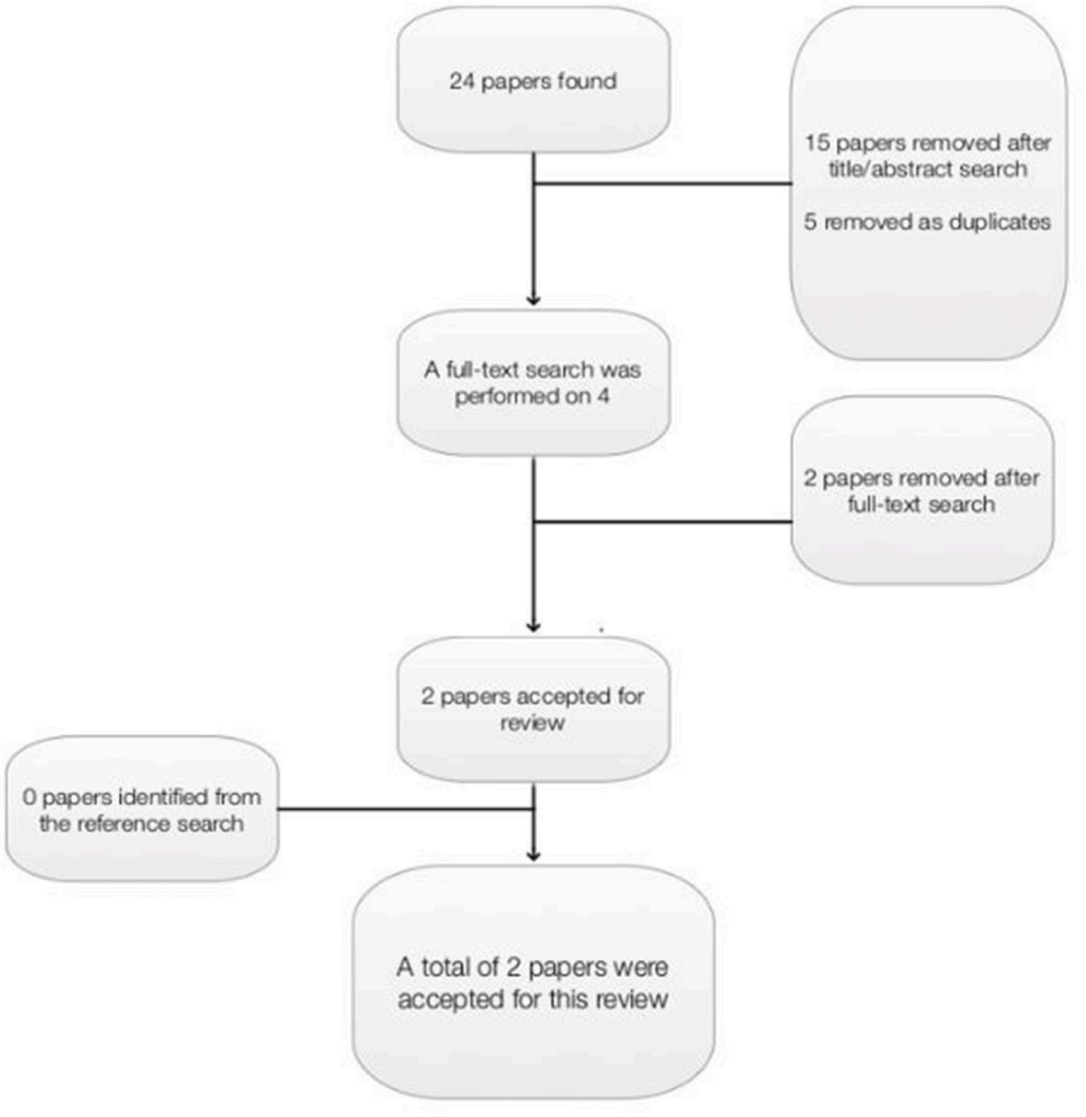
Papers retrieved as part of first systematic search.

The Critical Appraisal Skills Programme tool (CASP, 2017a,b) for systematic reviews influenced the search strategy and was used to determine the quality of papers, and only those deemed of medium-high quality were included for review. Papers were excluded if they were not written in English, they reviewed non-Randomized-Controlled Trials (RCTs), they were not peer-reviewed, the review included RCTs including only children or adolescents, or EMDR therapy was not the focus of the report. A wildcard search strategy was utilized, to ensure that relevant papers were not excluded based on international spelling variations. A total of 24 papers were retrieved from the database search: ASSIA 2; CINAHL 2; Cochrane library 4; Medline 6; Psyc Articles 1; PubMed 0; Science Direct 1; Web of Science 8 (Figure 2). Fifteen papers were removed after an initial title and abstract search, and five papers were removed as duplicates. Four papers were read in full, and two papers were further removed as one was not written in English, and one involved children and adolescents only. A reference and citation search was conducted on all relevant papers to maximize the identification of relevant studies, however, no further papers were included as a result of this. A total of two papers were included in this review (Table 2).

**Table 2:** Characteristics of papers included in the first systematic search.

**Author(s)**	**Aim**	**Design**	**Studies included (*n* =)**	**Total participants included (*n* =)**	**RCT quality assessment**	**Homogeneity measured**	**Publication bias**	**Effect size calculation**	**Location**
Chen et al. (2014)	To examine the effects of EMDR on symptoms of PTSD, depression, anxiety, or subjective distress in PTSD patients	Meta-analysis	26	1,133	RCT requirements met by Cochrane collaboration (Higgins and Green, 2011)	Yes	Funnel plot Egger's test (Egger et al., 1997)	Hedge's *g* Cohen's *d*	Taiwan
Chen et al. (2015)	To examine the efficacy of EMDR compared to CBT for adults with PTSD	Meta-analysis	11	424	RCT requirements met by Cochrane collaboration (Higgins and Green, 2011)	Yes	Funnel plot Begg's test (Begg and Mazumdar, 1994) Egger's test (Egger et al., 1997)	Standard Mean Difference	China

### Search 2

Search 2 aimed to examine the evidence underpinning the use of EMDR as a form of therapy that has been published since 2014. All databases, search fields, language and exclusion criteria were identical to those search 1, however search terms and year of publication differed (Table 3). All databases were accessed using Northumbria University library's online subscription.

**Table 3:** Search strategy utilized as part of second systematic search.

Search terms	(eye movement desensitization reprocessing OR EMDR) AND (randomized controlled trial OR RCT) AND (post-traumatic stress disorder OR PTSD)
Year of publication	January 2014-April 2017

**Figure 3:**
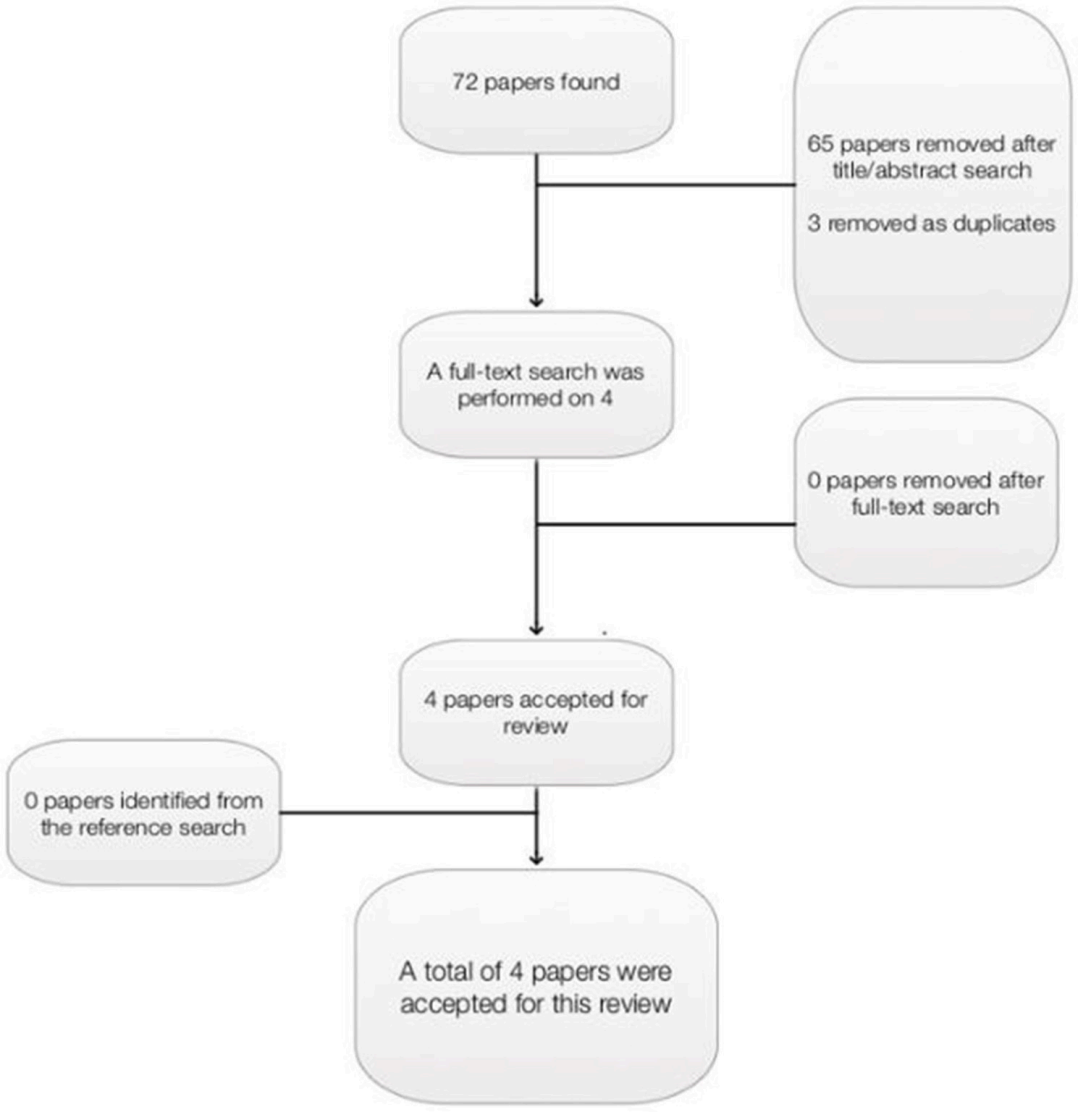
Papers retrieved as part of second systematic search.

The most recent meta-analysis included evidence prior to 2014, therefore it is imperative that studies between 2014 and 2017 are also considered. A second database search was therefore carried out, considering RCT evidence of studies examining the efficacy of EMDR therapy on PTSD symptoms between January 2014 and April 2017. As with search 1 papers were excluded if they were not written in English, they were not RCTs, they were not peer-reviewed, they were a pilot study or reported protocol data, they involved only children/adolescents under 18 years old, or EMDR therapy was not the focus of the report. A wildcard search strategy was utilized, to ensure that relevant papers were not excluded based on international spelling variations. Again, the Critical Appraisal Skills Programme tool (CASP, 2017a,b) for RCT evidence was used to determine the quality of papers, and papers were excluded if they did not satisfy CASP criteria. A total of 72 papers were retrieved from the database search: ASSIA 4; CINAHL 1; Medline 5; Psyc Articles 2; PubMed 3; Science Direct 10; Web of Science 47 (Figure 3).

Sixty-five papers were removed after an initial title and abstract search, and three papers were removed as duplicates. Four papers were read in full. A reference and citation search was conducted on all relevant papers to maximize the identification of relevant studies, however no further papers were included as a result of this. A total of four papers were included in this review (Table 4).

**Table 4:** Characteristics of papers included in the second systematic search.

**Author(s)**	**Aim**	**Sample**	**Intervention (participants)**	**Randomization**	**Blindness**	**Power analyses**	**Drop-out rate**	**Outcome measures**	**Time of assessment**	**Location**
Acarturk et al. (2016)	To examine effect of EMDR on symptoms of PTSD and depression in Syrian refugees	70 adult Syrian refugees with a PTSD diagnosis	EMDR (*n* = 37) Waiting list (*n* = 33) No further information provided	Randomly allocated to treatment but no details given	Blind to outcome assessor	Not discussed	EMDR (*n* = 12) Waiting list (*n* = 16) Non-significant across conditions	BDI-II IES-R HTQ HSCL-25 M.I.N.I	Pre-treatment 1-week post-treatment 5-weeks follow up	Turkey
Carletto et al. (2016)	To examine usefulness of EMDR and relaxation therapy as treatment for PTSD in patients with multiple sclerosis	50 adults diagnosed with multiple sclerosis	Ten 60-min EMDR sessions (*n* = 20) Ten 60-min sessions of relaxation therapy (*n* = 22)	Randomly allocated in 1:1 ratio	Blind to outcome assessor	Not discussed	EMDR (*n* = 5) Relaxation therapy (*n* = 3)	CMDI EDSS FAMS FSS HADS TAQ	Pre-treatment 12-15 weeks post-treatment 6-month follow up	Italy
de Bont et al. (2016)	To examine effects of prolonged exposure and EMDR for symptoms of PTSD in patients with chronic psychotic disorders	155 adults with chronic psychotic disorders	Eight EMDR sessions (*n* = 55) Eight sessions of prolonged exposure (*n* = 53) Waiting list (*n* = 47)	Randomly allocated to treatment but no details given	Blind to outcome assessor	Not discussed	EMDR (*n* = 11) Prolonged exposure (*n* = 6) Waiting list (*n* = 8)	BDI-II PRTS PSP PSYRATS SCI-SR-PANSS	Pre-treatment Post-treatment 6-month follow up	The Netherlands
ter Heide et al. (2016)	To examine safety and efficacy if EMDR in adult refugees with PTSD	72 adult refugees diagnosed with PTSD	Three 60-min; six 90-min EMDR sessions (*n* = 43) Twelve 60-min stabilization sessions (*n* = 45)	Blocked, simple randomization using a flipped coin	Blind to outcome assessor	80% power to detect medium effect size	EMDR (*n* = 7) Stabilization (*n* = 9) Non-significant across conditions	BREF CAPS HSCL HTQ WHOQOL-	Pre-treatment 2 weeks post-treatment 3-month follow up	The Netherlands

## Results

### Search 1

Two meta-analyses were included in this review (Chen et al., 2014, 2015). One was carried out in Taiwan (Chen et al., 2014) and one was carried out in China (Chen et al., 2015). One review focused on the use of EMDR therapy for adults with PTSD (Chen et al., 2015), whereas, one review included studies with both adults and children (5 of 26 RCTS involved children) (Chen et al., 2014). One meta-analysis focused on the efficacy of EMDR therapy compared to various interventions and control conditions (Chen et al., 2014) whereas, one study specifically focused on the efficacy of EMDR compared to CBT (Chen et al., 2015). Although this meta-analysis specifically compared EMDR therapy to CBT, many variants of CBT were included: image habituation training, trauma-treatment protocol, exposure plus cognitive reconstruction, prolonged exposure, stress inoculation training with prolonged exposure, imaginal exposure, brief eclectic psychotherapies, and “less standardized” CBT (Chen et al., 2015). Neither meta-analysis reported the length of follow-up for RCTs (Chen et al., 2014, 2015).

A total of 37 RCTs, and 1557 participants, were included over both meta-analyses. A total of seven RCTs were included in both of the reviews. It is evident that a vast number of comparator interventions and control conditions were used as comparisons to EMDR therapy. Furthermore, it is clear that there are severe inconsistencies between the outcome measures used to assess symptoms of PTSD, anxiety and depression, among other symptoms. Inconsistences also persist in use of scale sub-sections, as well as the scale version used.

Both meta-analyses followed PRISMA reporting guidelines (Chen et al., 2014, 2015). Meta-analyses provided in-depth, transparent evidence of their systematic search strategy. When examining the quality of RCTs, both studies utilized the Cochrane collaboration tool (Higgins and Green, 2011). The guidelines stipulate that a research quality score of 6–10 indicates an acceptable level of quality. One meta-analysis did not give quality indicators but described the quality assessment process (Chen et al., 2015), whereas, one meta-analysis stated that research quality of RCTs varied from 6 to 8 (Chen et al., 2014). Homogeneity among studies was measured in both meta-analyses (Chen et al., 2014, 2015) and publication bias was measured using funnel plot (Chen et al., 2014, 2015), Egger's test (Chen et al., 2014, 2015), and Begg's test (Chen et al., 2015). One study calculated effect size using Hedge's *g* and Cohen's *d* (Chen et al., 2014), and one study calculated effect size using Standard Mean Difference (Chen et al., 2015).

Both meta-analyses reported EMDR therapy as being significantly more effective in reducing PTSD symptoms than control conditions and other interventions, including CBT. Chen et al. (2014) conducted a meta-analysis specifically looking at the efficacy of EMDR therapy on the symptoms of PTSD (Chen et al., 2014). Twenty-two of the 26 studies examined the effect of EMDR therapy on PTSD symptoms. The meta-analysis data reported that EMDR therapy significantly reduced PTSD symptoms overall (*p* < 0.001), with moderate effects sizes being evident (*g* = −0.662). In this instance, there were no reported publication biases, however, substantial heterogeneity was reported between studies.

Similarly, within the meta-analysis conducted by Chen et al. (2015) examining the efficacy of EMDR therapy to CBT, EMDR therapy was determined as being significantly more effective than CBT in reducing PTSD symptoms (*p* = 0.05)(Chen et al., 2015). No publication bias was reported, however, heterogeneity was high. Focusing on sub-scales of PTSD, EMDR therapy was also significantly more beneficial than CBT in reducing severity of intrusion (*p* = 0.02) and arousal (*p* = 0.04) (Chen et al., 2015). Only symptoms of avoidance failed to show a significant difference, and both EMDR therapy and CBT were comparable for this outcome (*p* = 0.1) (Chen et al., 2015). No publication bias was reported, however, heterogeneity ranged from moderate to high on all three sub-scales.

Further analyses within the meta-analysis carried out by Chen et al. (2014) revealed that group therapy carried out with experienced therapists showed a significantly larger effect size on PTSD symptoms than when carried out with an inexperienced therapist (*g* = −0.753; *g* = −0.234, respectively; *p* = 0.007)(Chen et al., 2014).

Chen et al. (2014) also investigated the efficacy of EMDR therapy on symptoms of depression and anxiety (Chen et al., 2014). Twenty of the 25 RCTs examined the effect of EMDR therapy on symptoms of depression, as the primary outcome. Findings from the meta-analysis report EMDR therapy as significantly reducing symptoms of depression overall (*p* < 0.001), with moderate effects being evident (*g* = −0.643) (Chen et al., 2014). Once more, no publication bias was reported, however, heterogeneity was moderate.

Sixteen of the 26 RCTs within the meta-analysis carried out by Chen et al. (2014) measured symptoms of anxiety as a primary outcome (Chen et al., 2014). EMDR therapy significantly reduced symptoms of anxiety (*p* < 0.001) with a moderate effect size being evident (*g* = −0.640)(Chen et al., 2014). No publication bias was reported, but heterogeneity was moderate. Finally, 12 of the 26 RCTs within the meta-analysis conducted by Chen et al. (2014) reported a significant reduction of subjective distress (*p* < 0.01) (Chen et al., 2014). A large effect size was evident illustrating the efficacy of EMDR therapy on subjective distress (*g* = −0.956) (Chen et al., 2014). Once more, no publication bias was reported but heterogeneity was moderate to high.

Chen et al. (2014) further reported that longer treatment sessions, of more than 60 min, were significantly more effective than shorter sessions for symptoms of depression (*p* = 0.007) and were also significantly more effective for symptoms of anxiety (*p* = 0.045). In this instance, homogeneity was reported over studies.

#### Summary search 1

Both meta-analyses demonstrated the efficacy of EMDR therapy in treating symptoms of PTSD. Both studies concluded that EMDR therapy was more effective in treating symptoms of PTSD than various interventions and control conditions (Chen et al., 2014), including forms of CBT (Chen et al., 2015). Furthermore, Chen et al. (2014) demonstrated that EMDR therapy significantly reduced symptoms of depression, anxiety, and subjective distress (Chen et al., 2014). Chen et al. (2014) extrapolated further factors from RCT findings to determine that therapist experience of group therapy was a factor in reducing symptoms of PTSD. The meta-analysis identified that treatments lasting more than 60 min per session was a factor in improving symptoms of depression and anxiety (Chen et al., 2014).

There are however limitations to these studies. Both meta-analyses acknowledge that there is a lack of homogeneity between the RCTs reviewed, as variances exist between study design, interventions or control conditions used (including variations of CBT), sample sizes, and outcome measures including the use of various sub-scales or versions. The differences in study characteristics compromise the conclusions carried forward from these studies. Furthermore, one meta-analysis compares the efficacy of EMDR therapy to other interventions and control conditions, however, does not distinguish the differences of efficacy between these groups (Chen et al., 2014).

### Search 2

All studies examined the efficacy of EMDR therapy with individuals diagnosed with PTSD (Acarturk et al., 2016; Carletto et al., 2016; de Bont et al., 2016; ter Heide et al., 2016), with all but one study examining the impact of EMDR therapy on symptoms of PTSD (Acarturk et al., 2016; Carletto et al., 2016; ter Heide et al., 2016). Two studies examined the use of EMDR therapy with refugees diagnosed with PTSD (Acarturk et al., 2016; ter Heide et al., 2016), one study examined the use of EMDR therapy for symptoms of PTSD in patients diagnosed with multiple sclerosis (Carletto et al., 2016), and one study looked at effect of PTSD, depression and social functioning in patients with chronic psychotic disorders (de Bont et al., 2016). All studies used EMDR therapy as the intervention (Acarturk et al., 2016; Carletto et al., 2016; de Bont et al., 2016; ter Heide et al., 2016). Two studies used additional intervention therapies; prolonged exposure (de Bont et al., 2016) and relaxation therapy (Carletto et al., 2016). Two studies included a waiting list group as a control measure (Acarturk et al., 2016; de Bont et al., 2016) and one study utilized stabilization as a control measure (ter Heide et al., 2016).

The number, and length, of sessions differed over the studies. One study did not provide details of treatment sessions (Acarturk et al., 2016), one study provided ten 60-min sessions (Carletto et al., 2016), one study provided eight sessions but provided no further detail (de Bont et al., 2016), and one study provided three 60-min sessions, followed by six 90-min sessions (ter Heide et al., 2016). Studies included between 50 and 155 participants (Acarturk et al., 2016; Carletto et al., 2016; de Bont et al., 2016; ter Heide et al., 2016) and all studies reported a low dropout rate, with two of these studies reporting non-significant difference across conditions (Acarturk et al., 2016; ter Heide et al., 2016). All studies randomized participants to treatment groups (Acarturk et al., 2016; Carletto et al., 2016; de Bont et al., 2016; ter Heide et al., 2016). In all studies, the treatment groups were blind to the assessor only (Acarturk et al., 2016; Carletto et al., 2016; de Bont et al., 2016; ter Heide et al., 2016) as EMDR therapy is a healthcare treatment administered by a professional, therefore a blind or double blind study is inappropriate.

Only one study described power analyses, and indicated 80% power to detect medium effect size (ter Heide et al., 2016). All studies utilized different outcome measures to report symptoms of PTSD, depression, anxiety, and others, with 19 different measures being used of the four studies. The time of assessment, and follow-up, also differed between the studies. All studies reported pre-test measures, post-test measures were carried out between 1 and 12/15 weeks post-test, and follow-up also varied between 5 weeks to 6 months post-intervention. One study was carried out in Turkey (Acarturk et al., 2016), one was carried out in Italy (Carletto et al., 2016), and two were carried out in the Netherlands (de Bont et al., 2016; ter Heide et al., 2016).

All three studies directly measuring symptoms of PTSD found EMDR therapy significantly improved these symptoms (Acarturk et al., 2016; Carletto et al., 2016; ter Heide et al., 2016). One study reported EMDR therapy as being significantly more effective than another intervention therapy (Carletto et al., 2016), one reported EMDR therapy as being significantly more effective than a waiting list control-group (Acarturk et al., 2016), and one study found EMDR therapy to significantly improve some PTSD symptoms, but no more than a stabilization control group (ter Heide et al., 2016).

Carletto et al. (2016) utilized both EMDR therapy and relaxation therapy as intervention therapies to reduce PTSD symptoms of individuals diagnosed with multiple sclerosis (Carletto et al., 2016). The study determined that 17 of 20 EMDR therapy participants no longer met PTSD diagnosis 12–15 weeks after treatment, and none of these 20 EMDR therapy participants met PTSD diagnosis at 6-month follow-up assessment. EMDR therapy was significantly more effective than relaxation therapy when considering post-treatment PTSD diagnosis (*p* = 0.049) (Carletto et al., 2016).

Acarturk et al. (2016) also concluded that EMDR therapy significantly reduced post-test PTSD diagnosis, compared to a waiting list control group (*p* < 0.01) (Acarturk et al., 2016). The study examined the efficacy of EMDR therapy for PTSD and depression among Syrian refugees. The results indicated that individuals in the waiting-list control group were 24.21 times more likely to be diagnosed with PTSD immediately post-test, compared to participants in the EMDR therapy group. Furthermore, the reduced likelihood of PTSD diagnosis remained significant at 1-month follow up, with individuals in the waiting-list control group being 23 times more likely to be diagnosed with PTSD, compared to EMDR therapy participants (*p* < 0.01)(Acarturk et al., 2016). Further analyses carried out by Acarturk et al. (2016) found EMDR therapy to significantly reduce the severity of PTSD compared to the waiting list control group (*p* < 0.001) and this effect was maintained over time. Specifically, there was a significant difference between EMDR therapy and control group for avoidance (*p* < 0.01), intrusion (*p* < 0.01), and hyper-arousal (*p* < 0.01). EMDR therapy also significantly improved reports of exposure of traumatic events compared to the control group condition (*p* < 0.01), and once more, this effect was maintained over time (Acarturk et al., 2016).

Similar to the study carried out by Acarturk et al. (2016), ter Heide et al. (2016) examined the efficacy of EMDR therapy for refugees diagnosed with PTSD (ter Heide et al., 2016). However, results were not as promising for the use of EMDR therapy in comparison. Over all of the reported primary and secondary outcomes, ter Heide et al. (2016) only reported significant improvement of trauma symptoms for both EMDR therapy and the stabilization control group (*p* < 0.05; *p* < 0.05), with no significant differences being reported between these conditions (ter Heide et al., 2016).

All four RCTs also considered the efficacy of EMDR therapy on symptoms of depression (Acarturk et al., 2016; Carletto et al., 2016; de Bont et al., 2016; ter Heide et al., 2016), and three of these also considered its efficacy on symptoms of anxiety (Acarturk et al., 2016; Carletto et al., 2016; ter Heide et al., 2016). Carletto et al. (2016) identified that both EMDR therapy and relaxation therapy significantly improved anxiety symptoms (*p* < 0.001), depressive symptoms (*p* < 0.001) and mood (*p* < 0.001), although there were no significant difference between treatment efficacy (Carletto et al., 2016). EMDR therapy was also determined as being effective in reducing symptoms of depression and anxiety in the study carried out by Acarturk et al. (2016) (Acarturk et al., 2016). The study reported a significant difference between EMDR therapy intervention group and a waiting-list control group for the symptoms of depression (*p* < 0.01) and anxiety (*p* < 0.01), with both effects being maintained over time.

Although de Bont et al. (2016) utilized EMDR therapy as a treatment for individuals diagnosed with PTSD, the RCT did not report PTSD symptoms as an outcome measure (de Bont et al., 2016). Instead, de Bont et al. (2016) looked at the effect of EMDR therapy on symptoms of psychosis, depression and social functioning. The results presented by de Bont et al. (2016) are less favorable for the efficacy of EMDR therapy than other studies. The study reported prolonged exposure as being significantly more effective in reducing symptoms of depression than EMDR therapy (de Bont et al., 2016). The study showed that depressive symptoms for those in the prolonged exposure intervention, were significantly reduced compared to participants in a waiting-list control group at all follow-up points, and to EMDR therapy (*p* < 0.05) at both 6 month follow-up and over time (de Bont et al., 2016). Similarly, ter Heide et al. (2016) did not report statistically significant differences for symptoms of either depression or anxiety either over time, or between EMDR therapy and the stabilization control group (ter Heide et al., 2016).

Other outcome measures were also considered within these RCTs; paranoid thoughts (de Bont et al., 2016), social functioning (de Bont et al., 2016), functional assessment (Carletto et al., 2016), fatigue (Carletto et al., 2016), and quality of life (ter Heide et al., 2016). In addition to symptoms of depression, de Bont et al.'s (2016) main outcome measures were symptoms of psychosis and social functioning. This study demonstrated the impact of prolonged therapy exposure and EMDR therapy in reducing psychotic symptoms over the waiting list control condition (de Bont et al., 2016). EMDR therapy significantly reduced paranoid thoughts post-treatment (*p* < 0.05) and over time (*p* < 0.05), but interestingly not at 6-month follow up. Prolonged exposure was also significantly more effective in reducing paranoid thoughts compared to waiting list controls (*p* < 0.05) at all follow-up points. Neither EMDR therapy nor prolonged exposure significantly impacted auditory hallucinations or personal social performance compared to waiting list control group (de Bont et al., 2016). Carletto et al. (2016) also assessed the impact of EMDR therapy, and relaxation therapy, on functional assessment (*p* = 0.001) and fatigue severity (*p* = 0.029). Although both EMDR therapy and relaxation therapy were effective in improving these symptoms, there were no significant differences between reported between treatment groups (Carletto et al., 2016). ter Heide et al. (2016) examined quality of life, however, like other findings from this study, there were no significant outcomes for the efficacy of EMDR therapy, or for effects between the EMDR therapy intervention group, and the stabilization control group (ter Heide et al., 2016).

#### Summary search 2

Four RCTs have been published between 2014 and 2017 examining the efficacy of EMDR therapy for individuals diagnosed with PTSD (Acarturk et al., 2016; Carletto et al., 2016; de Bont et al., 2016; ter Heide et al., 2016). EMDR therapy was reported as significantly improving PTSD diagnosis and PTSD symptoms, over time, compared to relaxation therapy and a waiting-list control group (Acarturk et al., 2016; Carletto et al., 2016). EMDR therapy was also reported as significantly improving trauma symptoms (ter Heide et al., 2016).

All four RCTs also measured symptoms of depression and anxiety. EMDR therapy was reported as significantly reducing both depression and anxiety (Acarturk et al., 2016; Carletto et al., 2016). This effect was significant compared to control group (Acarturk et al., 2016) but there were no significant differences reported between EMDR therapy and relation therapy in reducing these symptoms (Carletto et al., 2016). Contradictory to this, one study did not report any differences in depression or anxiety symptoms between EMDR therapy and stabilization control group (ter Heide et al., 2016), and one study reported prolonged exposure as being significantly more effective in reducing symptoms of depression than EMDR therapy and waiting-list control group at post-test and over time (de Bont et al., 2016).

Finally, EMDR therapy and prolonged exposure therapies were reported as being an effective therapy to improve paranoid thoughts both at post-treatment assessment and over time (de Bont et al., 2016), but had not impact on auditory hallucinations or personal social performance compared to a waiting-list control group. Both EMDR therapy and relaxation therapy significantly improved functional assessment and fatigue severity (Carletto et al., 2016), however EMDR therapy was not effective in improving quality of life compared to a control stabilization group (ter Heide et al., 2016).

Study limitations were present. Similar to the meta-analyses reviewed, there was a lack of homogeneity across study design, intervention, control, outcome measures, and follow-up procedures. This makes it difficult to synthesize findings across studies, and reduces the impact of conclusions derived from the evidence. Furthermore, only one of the four studies reported power analyses which reduces the impact of the findings. Finally, only two of the four studies followed up at 6 months, therefore restricting the evidence of impact over time.

## Discussion

EMDR therapy is an empirically validated form of Psychotherapy (Shapiro, 2014), recommended by the World Health Organization to treat trauma (World Health Organisation, 2013). Meta-analysis and RCT data within this review evidence the efficacy of EMDR therapy in primarily treating symptoms of PTSD, depression and anxiety. Studies covered a wide range of counties including East and West affirming the effective delivery of EMDR therapy to differing cultures (Acarturk et al., 2016; Carletto et al., 2016; de Bont et al., 2016; ter Heide et al., 2016). EMDR therapy significantly improved PTSD diagnosis (Carletto et al., 2016), and significantly reduced symptoms of PTSD (Chen et al., 2014, 2015; Acarturk et al., 2016; Carletto et al., 2016), and other trauma symptoms (ter Heide et al., 2016). Specifically, this review also evidenced EMDR therapy as significantly reducing symptoms of depression (Chen et al., 2014; Acarturk et al., 2016; Carletto et al., 2016), anxiety (Chen et al., 2014; Acarturk et al., 2016; Carletto et al., 2016), subjective distress (Chen et al., 2014), paranoid thoughts (de Bont et al., 2016), functional assessment (Carletto et al., 2016), and severe fatigue (Carletto et al., 2016). Despite the variations in methodology and analysis, the meta-analyses found EMDR therapy more effective than comparative interventions and control groups (Chen et al., 2014), resulting in PTSD below clinically significant levels. EMDR therapy was, however, more effective when delivered by more experienced therapists (Chen et al., 2015) and when sessions lasted more than 60 min (Chen et al., 2014). Overall, EMDR therapy was effective with a range of presenting problems and symptoms (Acarturk et al., 2016; Carletto et al., 2016; de Bont et al., 2016; ter Heide et al., 2016). Low drop-out rates across all studies indicates EMDR therapy is well tolerated by clients, including in comparison to prolonged exposure (Ironson et al., 2002; Evans, 2003; Bisson and Andrew, 2013; World Health Organisation, 2013; Shapiro, 2014; Acarturk et al., 2016; Carletto et al., 2016; de Bont et al., 2016; ter Heide et al., 2016). There were methodological limitations of the studies, which compromises the quality of data examined in this review. Initially, many of the RCT studies were low-powered due to small sample sizes used. Furthermore, studies reported limited follow-up data, and follow-up data that was reported was often differed between studies, limiting evidence of long-term efficacy. These limitations have been reported in other meta-analytic evidence examining PTSD therapies more widely, and it was acknowledged that these issues similarly hindered conclusions derived from the synthesized evidence (Bisson and Andrew, 2013).

Another limitation of the evidence to date is the lack of homogeneity between RCT evidence, due to the inconsistencies in study design, intervention characteristics, sample, outcome measures and follow-up procedures in each study. This lack of homogeneity limits comparability between data, and ultimately impacts conclusions. Furthermore, none of the retrieved studies reported economic factors of EMDR therapy, and this is seldom reported in wider EMDR therapy literature. It is acknowledged that EMDR therapy can reduce healthcare costs, whilst maintaining patient care, due to substantial patient improvement in relatively short time periods (Shapiro, 2014). However, evidence is required to examine these economic factors, specifically in comparison to similar therapies such as CBT.

### Search limitations

A strength of the review is that all papers were reviewed using the Critical Appraisal Skills Programme (CASP) tools for systematic reviews or RCTs, and studies were not included if they did not meet CASP criterion. It is also acknowledged that this review is limited to RCT evidence specifically of adults receiving EMDR therapy, a specific population with definite characteristics, and therefore findings cannot be more widely generalized. There were some limitations to the first literature search. Only meta-analyses and systematic searches with, EMDR, in their title were included as part of the first search. This was due to the refinement of the search strategy, which initially included syntheses of multiple forms of therapy. However, by including evidence looking at multiple forms of therapy, some syntheses included only one or two studies investigating EMDR therapy, and often did not specifically analyse the efficacy of EMDR therapy as a stand-alone treatment. Therefore, limited evidence could be retrieved from these papers, and a decision was made to only examine papers directly investigating the efficacy of EMDR therapy. The second systematic search examined RCT evidence only as RCT evidence is considered gold standard evidence for the efficacy of healthcare interventions (Evans, 2003), and alternative evidence was therefore excluded from this report.

## Conclusion

As the global burden of psychological trauma continues unabated, the need for more research and investigation into treatment interventions that are both effective and efficient is essential. It is clear from this extensive, robust evidence that EMDR therapy is an effective treatment to improve diagnosis of PTSD, and reduce symptoms of PTSD, and other trauma-related symptoms. More RCT evidence is required to further enhance our collective understanding of PTSD and co-morbid symptoms.

### Recommendations for practice

EMDR therapy should be available for adults who present with PTSD and co-morbid symptoms including depression and anxiety and EMDR therapy can be delivered effectively within the countries identified within this study.

### Recommendations for future research

Further RCTs of EMDR therapy with larger sample sizes are required with a wide range of presenting mental health problems.

Additional research examining the differences between adult and child PTSD to ascertain which psychological treatment approaches for children and adolescents are more effective and efficient, as current evidence is weak. However emerging Practice-Based Evidence increasingly supports the utilization of Group Trauma Treatment Interventions (Jarero et al., 2013).

- More standardization of the normative outcome measures is required to facilitate comparison across studies.
- Studies need to include longitudinal evaluation beyond 6 months.
- Analysis is required of the economic benefits of EMDR therapy in comparison with other trauma-focused interventions.
- Comparative studies are needed of the efficacy of EMDR therapy across cultures.

## Author contributions

GW carried out the systematic searches and synthesized the evidence. All authors contributed to the writing and editing of the paper. All authors approved the final manuscript.

### Conflict of interest statement

The authors declare that the research was conducted in the absence of any commercial or financial relationships that could be construed as a potential conflict of interest.
